# δ‐Catenin regulates proliferation and apoptosis in renal cell carcinoma via promoting β‐catenin nuclear localization and activating its downstream target genes

**DOI:** 10.1002/cam4.2857

**Published:** 2020-01-28

**Authors:** Lincheng Ju, Liping Shan, Bo Yin, Yongsheng Song

**Affiliations:** ^1^ Department of Urology Shengjing Hospital of China Medical University Shenyang China

**Keywords:** δ‐catenin, renal cell carcinoma, β‐catenin, proliferation, apoptosis

## Abstract

δ‐Catenin is a unique member of the catenin family and is proved to be overexpressed in diverse human cancer types. However, the clinical significance and underling mechanism of δ‐catenin expression in renal cell carcinoma (RCC) remain elusive. Herein, we detected the protein expression of δ‐catenin in 28 clinical specimens of paired renal cancer tissues and normal renal tissues by Western blot analysis. δ‐Catenin expression in 58 cases of renal cell carcinoma was also examined by immunohistochemistry, and its association with clinicopathological factors was analyzed by statistical analysis. In vitro and in vivo assays were employed to further explore the biological role of δ‐catenin in RCC. The results showed that δ‐catenin was highly expressed in both clinical samples and cell lines of RCC. RCC patients with higher δ‐catenin expression had a more advanced pTNM stage and tumor stage as well as lymph nodes metastasis than those with lower expression. By regulating the nuclear translocation of β‐catenin and β‐catenin‐mediated oncogenic signals, δ‐catenin promoted proliferation and inhibited apoptosis in RCC. In vivo assay indicated δ‐catenin facilitated tumor growth in ACHN cell xenograft mouse model. Taken together, our study suggests that δ‐catenin might be considered as a novel prognostic indicator and actionable target for gene therapy in renal cell carcinoma.

## Graphical abstract

By regulating the nuclear translocation of β‐catenin and β‐catenin‐mediated oncogenic signals, δ‐catenin promoted proliferation and inhibited apoptosis of renal cancer cells.

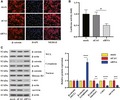

## 1 INTRODUCTION

Renal cell carcinoma (RCC) is the ninth most common malignancy, with 403 262 new cases diagnosed and 175 098 cancer deaths worldwide in 2018.^1^, ^2^ In the management of localized or locally advanced RCC, surgical resection including partial and radical nephrectomy is a common treatment strategy.^3^, ^4^ However, up to 40% risk of developing recurrence after nephrectomy.^4^, ^5^ For patients with metastatic RCC, cytoreductive nephrectomy followed by systemic drugs is an established treatment approach.^6^, ^7^ Nevertheless, the disease still progresses after therapy and metastasis is the major cause of their death.^1^ Therefore, treatments for the recurrence and metastasis of RCC are clearly needed to improve current therapies.

δ‐Catenin, alternatively known as CTNND2 or NPRAP (neural plakophilin‐related armadillo protein), is a member of the catenin family. It is the adhesive junction‐associated protein that was originally identified to be enriched in brain tissues.^8^, ^9^, ^10^, ^11^ Recent researches have heightened that δ‐catenin is also implicated in some cancers. It is evidenced that δ‐catenin facilitates the malignant phenotype of non‐small‐cell lung cancer through binding to the juxtamembrane domain (JMD) of E‐cadherin.^12^ Beyond this, two research articles indicated that ectopic overexpression of δ‐catenin is related to prostate cancer progression. One reported δ‐catenin causes the alteration of cell cycle and survival gene profiles. The other presented the mechanism that δ‐catenin induces E‐cadherin processing and activates β‐catenin‐mediated oncogenic signals.^13^, ^14^ To date, the precise role of δ‐catenin in tumorigenesis and development of RCC is still poorly understood, neither is the correlation between its expression and clinical pathological parameters. All these deserve to be explored.

β‐Catenin, a crucial member of the adherent junctions,^15^ is located at adhesion complexes at the cytoplasmic side of membrane in normal cells.^16^ β‐Catenin is phosphorylated in the cytoplasm by a multiprotein destruction complex, which leads to proteasome‐mediated degradation.^17^ In pathological conditions, due to mutations on β‐catenin sequence, blocking the activity of the destruction complex or preventing β‐catenin from being phosphorylated by GSK3‐3β, β‐catenin may stabilize and escape from its degradation fate.^18^ This results in elevation of cytoplasmic β‐catenin and its translocation to the nucleus. In the nucleus, β‐catenin interacts with transcription factors from the T‐cell factor (TCF)/Lymphoid enhancer‐binding factor (LEF) family,^19^, ^20^, ^21^ which triggers transcription of Wnt/β‐catenin target genes.^22^, ^23^ The aberrant activation of these genes involved in proliferation, apoptosis and invasion contributes to cancer progression.^24^, ^25^, ^26^ Moreover, it has been demonstrated that β‐catenin can be activated in RCC development,^27^, ^28^ suggesting a possible dysregulation of this protein. However, the regulation of β‐catenin by δ‐catenin and the corresponding mechanism have not been studied yet.

In the present study, we detected the expression of δ‐catenin in RCC specimens by Western blot analysis and immunohistochemistry staining, and then analyzed the correlation between its expression and clinicopathological factors. To gain insights into the role of δ‐catenin in RCC, we explored whether it was implicated in proliferation and apoptosis of RCC cells. In addition, the regulation of δ‐catenin on β‐catenin and β‐catenin‐mediated oncogenic signals were also investigated. Moreover, we determined the influence of δ‐catenin on tumor growth in the xenograft mouse model. Our results illuminated that elevated δ‐catenin expression in RCC caused the activation of β‐catenin and its target genes, thereby affecting proliferation and apoptosis. This cognition of δ‐catenin may help to establish a novel targeted therapy and improve current therapies of RCC.

## 2 MATERIALS AND METHODS

### 2.1 Cell lines and cell culture

Normal human renal epithelial cells (HK‐2) and human renal cancer cell lines (A498, ACHN and 786‐O) were obtained from Procell Life Science & Technology (Wuhan, China) and cultured in MEM medium or RPMI‐1640 medium supplemented with 10% fetal bovine serum (BI). All the cells were maintained in a humidified incubator with 5% CO2 at 37°C.

### 2.2 Nuclear and cytoplasm protein extraction

The cells were lysed in 200 μL cytoplasmic lysis buffer A supplemented with PMSF on ice for 10‐15 minutes, and then added with 10 μL cytoplasmic lysis buffer B. The mixture was vortexed for 5 seconds and centrifuged at 12 000 × *g* for 5 minutes at 4°C. The supernatant was a cytoplasmic extract. The pellet was resuspended with 50 μL nuclei lysis buffer (Beyotime, P0028) and kept in ice for 30 minutes (vortexed 15‐30 seconds at a high speed every 1‐2 minutes). Nuclear proteins were extracted by centrifugation at 12 000 × *g* for 10 minutes at 4°C. The supernatant was the nuclear extract. The protein concentration was assessed by Enhanced BCA Protein Assay Kit (Beyotime, P0009).

### 2.3 Western blot analysis

Cells were collected and lysed with RIPA lysis buffer (Beyotime, P0013B). The protein concentrations were evaluated via Enhanced BCA Protein Assay Kit (Beyotime). Protein lysates were separated by electrophoresis on a sodium dodecyl sulfate‐polyacrylamide gel (8%‐15%), transferred onto PVDF membranes, and incubated with primary antibodies at 4°C overnight followed by goat anti‐rabbit or goat anti‐mouse IgG (Proteintech) at 37°C for 40 minutes. The protein bands were visualized by enhanced chemiluminescence (ECL). Primary antibodies used in this study were as follows: anti‐δ‐Catenin antibody (Bioss, bs‐22251R), anti‐Cyclin D1 antibody (CST, #2922), anti‐CDK6 antibody (Proteintech, 19117‐1‐AP), anti‐CDK4 antibody (CST, #12790), anti‐c‐myc antibody (Proteintech, 10828‐1‐AP), anti‐Bcl2L1 antibody (Proteintech, 10783‐1‐AP), anti‐cleaved‐caspase‐3 antibody (CST, #9661), anti‐survivin antibody (Proteintech, 10508‐1‐AP), anti‐β‐catenin antibody (CST, #8480), anti‐β‐actin antibody (Proteintech, 60008‐1‐Ig), anti‐Histone H3 antibody (Proteintech, 17168‐1‐AP).

### 2.4 Plasmid and siRNA transfection

A498 and ACHN cells were transfected by δ‐Catenin‐specific siRNA (5ʹ‐CCCUAGCAGUACUGACCAATT‐3ʹ and 5ʹ‐UUGGUCAGUACUGCUAGGGTT‐3ʹ) or siCtrl (5ʹ‐UUCUCCGAACGUGUCACGUTT‐3ʹ and 5ʹ ‐ACGUGACACGUUCGGAGAATT‐3ʹ) encapsulated with Lipofectamine 2000 (Invitrogen, 11668‐019) following its transfection protocol. After 48 hours of transfection, subsequent assays were performed as described. ACHN cells were transfected by shRNA plasmids or shCtrl plasmids as the negative control with Lipofectamine 2000 for silencing δ‐catenin. After 24 hours, the cells were selected in media supplemented with 300 μg/mL G418 to obtain the stably transduced cells.

### 2.5 RNA extraction and quantitative real‐time PCR (qRT‐PCR)

Total RNA was isolated from RCC cells using total RNA rapid extraction kit (BioTeke, RP1201) according to the manufacturer's protocol. The obtained RNA was reverse transcribed into cDNA by M‐MLV (Takara, 2641A). The cDNA was used to perform real‐time PCR with Taq HS Perfect Mix (Takara, R300A) and SYBR Green (BioTeke, EP1602). The data were determined by Exicycler™ 96 (Bioneer, Daejeon, Korea), calculated by 2^−∆∆Ct^ parameters. Sequences of real‐time PCR primers were as follows: δ‐Catenin: forward: 5ʹ‐TCTGAGAAACCTGGTGTATGG‐3ʹ, reverse: 5ʹ‐CAGTCGTCTTGCGGAGTAA‐3ʹ, β‐actin: forward: 5ʹ‐CCACTGCCGCATCCTCTT‐3ʹ, reverse: 5ʹ‐GGTCTTTACGGATGTCAACG‐3ʹ.

### 2.6 CCK‐8 assay

Cell counting kit‐8 (CCK‐8) assay was employed to detect cell viability. RCC cells were seeded into 96‐well plates with 4 × 10^3^ per well beforehand. After being adhered, the cells were transfected with siCtrl and δ‐catenin siRNA; and CCK‐8 reagent (Sigma) was added into the 96‐well plates with 10 µL per well and incubated for 1 hour. The optical density (OD) value was measured at 450 nm with a microplate reader (BioTek). The OD450 values at 0, 24, 48, 72, and 96 hours were tested, respectively.

### 2.7 Immunofluorescence

The slides of cells were fixed for 15 minutes with 4% paraformaldehyde and permeabilized with 0.1% tritonX‐100 for 30 minutes. The samples were blocked by goat serum for 15 minutes. Primary antibody (diluted to 1:200 with PBS) such us Ki67 and β‐catenin was added to the cells and incubated overnight at 4°C. After PBS washing, cells were incubated with Cy3‐labeled goat anti‐rabbit IgG (Beyotime, A0516) diluted in PBS (1:200) for 1 hours at room temperature. The nuclei were counterstained by DAPI. Finally, the samples were sealed by anti‐fade reagent and observed using the OLUMPUS fluorescence microscope (at 400 × magnification).

### 2.8 Flow cytometry

Flow cytometry was performed to detect the cell cycle and apoptosis of RCC cells. After transfection for 48 hours, cells were collected, treated by Cell Cycle Analysis kit (Beyotime) following the manufacturer's protocol, and detected using NovoCyte flow cytometer (ACEA Biosciencs). For the apoptosis analysis, the cells were treated with Annexin V‐FITC Apoptosis Detection Kit (Beyotime) and accessed through flow cytometer.

### 2.9 TOP/FOP luciferase reporter assay

ACHN cells transfected with δ‐catenin siRNA or siCtrl were seeded in 6‐well plates. After incubation for 24 hours, the cells were transfected with 2 μg β‐catenin responsive firefly luciferase reporter plasmid TOPflash or negative control FOPflash and 0.5 μg constitutively active vector encoding Renilla luciferase using lipofectamine 2000. At 24 hours after transfection, the cells were lysed and then firefly and Renilla luciferase activities were determined using the dual luciferase reporter assay system (Promega) according to the manufacturer's instructions.

### 2.10 Animal studies

All animal experiments in this study were approved by the Institutional Animal Ethics Committee of China Medical University, and were carried according to the Guideline for the Care and Use of Laboratory Animals. Male Balb/c nude mice at the age of 6 weeks were divided into two groups randomly (6 mice per group). ACHN cells (1 × 10^7^) with stable knockdown of δ‐catenin with shRNA plasmids or ACHN cells transfected with shCtrl plasmids were injected subcutaneously into the right flank of the nude mice. The tumor size was measured every four days, and the tumor volume was calculated according to the following: *V* = (width^2^ × length)/2. The mice were sacrificed 27 days after injection. Tumors were excised and the tumor weight was recorded.

### 2.11 Immunohistochemistry staining assay

The slides were heated at 60°C for 2 hours, deparaffinized in xylene, and rehydrated by graded ethanol. After antigen retrieval, the tissue sections were blocked with goat serum. The sections were then exposed to antibodies against δ‐catenin or Ki67 (Abcam, ab15580) overnight at 4°C. The slides were washed with PBS and incubated with HRP‐labeled goat anti‐rabbit antibody (ThermoFisher, #31460) for 1 hour. After washing, the slides were added with diaminobenzidine (DAB) and counterstained with hematoxylin. In the end, the sections were dehydrated by graded ethanol, sealed by neutral balsam, and visualized with microscope (at 400 × magnification).

### 2.12 Statistical analysis

The data were presented as mean ± SD in the figures. Statistical analysis was performed with two‐tailed unpaired t‐test, one‐way ANOVA or two‐way ANOVA as indicated. *P* value < .05 was considered statistically significant.

## 3 RESULTS

### 3.1 δ‐Catenin was highly expressed in human renal cancer tissues and correlated with poor prognosis of RCC patients

**Figure 1:**
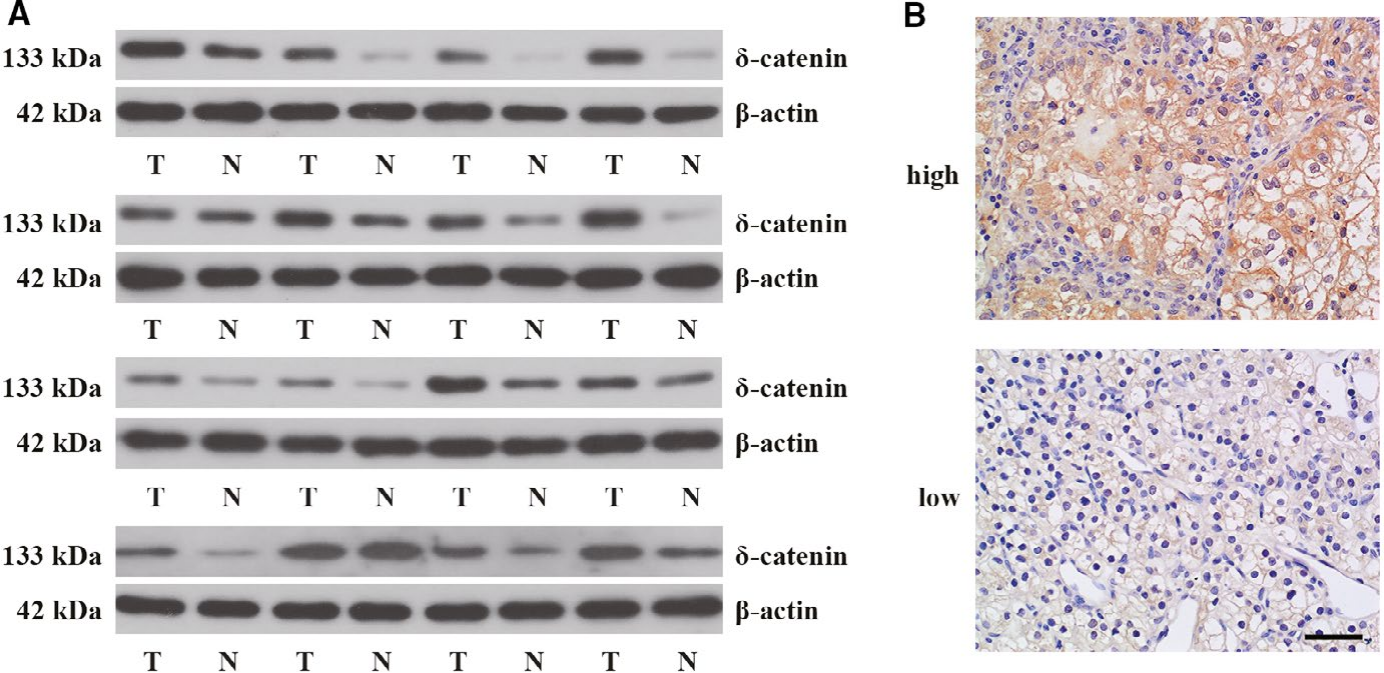
δ‐Catenin was highly expressed in human renal cancer tissues. A, The representative bands of δ‐catenin detected by Western blot analysis from human renal cancer tissues and adjacent normal tissues (16 pairs). B, The representative images of low and high expression of δ‐catenin in human renal cancer tissues visualized by immunohistochemical staining. Scale bar, 50 μm

We firstly determined the expression of δ‐catenin in human renal cancer tissues. Western blot analysis was performed to examine δ‐catenin expression from 28 cases of patients with RCC. The results showed that δ‐catenin was highly expressed in tumor tissues compared with adjacent normal tissues (Figure 1A and Figure S1A). Furthermore, we detected the expression of δ‐catenin by immunohistochemistry in 58 cases of RCC, and evaluated the correlation between δ‐catenin expression and clinicopathological factors. The representative images of low and high expression of δ‐catenin from patients were presented in Figure 1B. As shown in Table 1, high expression of δ‐catenin was correlated with pTNM stage, tumor stage, and lymph node metastasis (*P* < .05), but not correlated with patient's gender, age, tumor size, and distant metastasis (*P* > .05). These results together demonstrated that the expression of δ‐catenin was enhanced in RCC tissues, which was associated with poor prognosis in RCC patients.

**Table 1:** Relationship between δ‐catenin and clinical characteristics in RCC patients

Parameters	Group	δ‐catenin expression		*P* value
		High (n = 31)	Low (n = 27)	
Gender	Male	17	13	.80628
	Female	14	14	
Age	<60	20	14	.47797
	≥60	11	13	
Tumor size (cm)	<4 cm	1	3	.50751
	≥4 cm	30	24	
pTNM stage	I/II	21	27	**.00378**
	III/IV	10	0	
Tumor stage	T1‐T2	23	27	**.01384**
	T3‐T4	8	0	
Lymph nodes metastasis	Negative	25	27	**.04747**
	Positive	6	0	
Distant metastasis	Negative	29	27	.53404
	Positive	2	0	

A *P* value of less than .05 was considered to be statistically significant (shown in bold).

### 3.2 δ‐Catenin was highly expressed in human renal cancer cells

**Figure 2:**
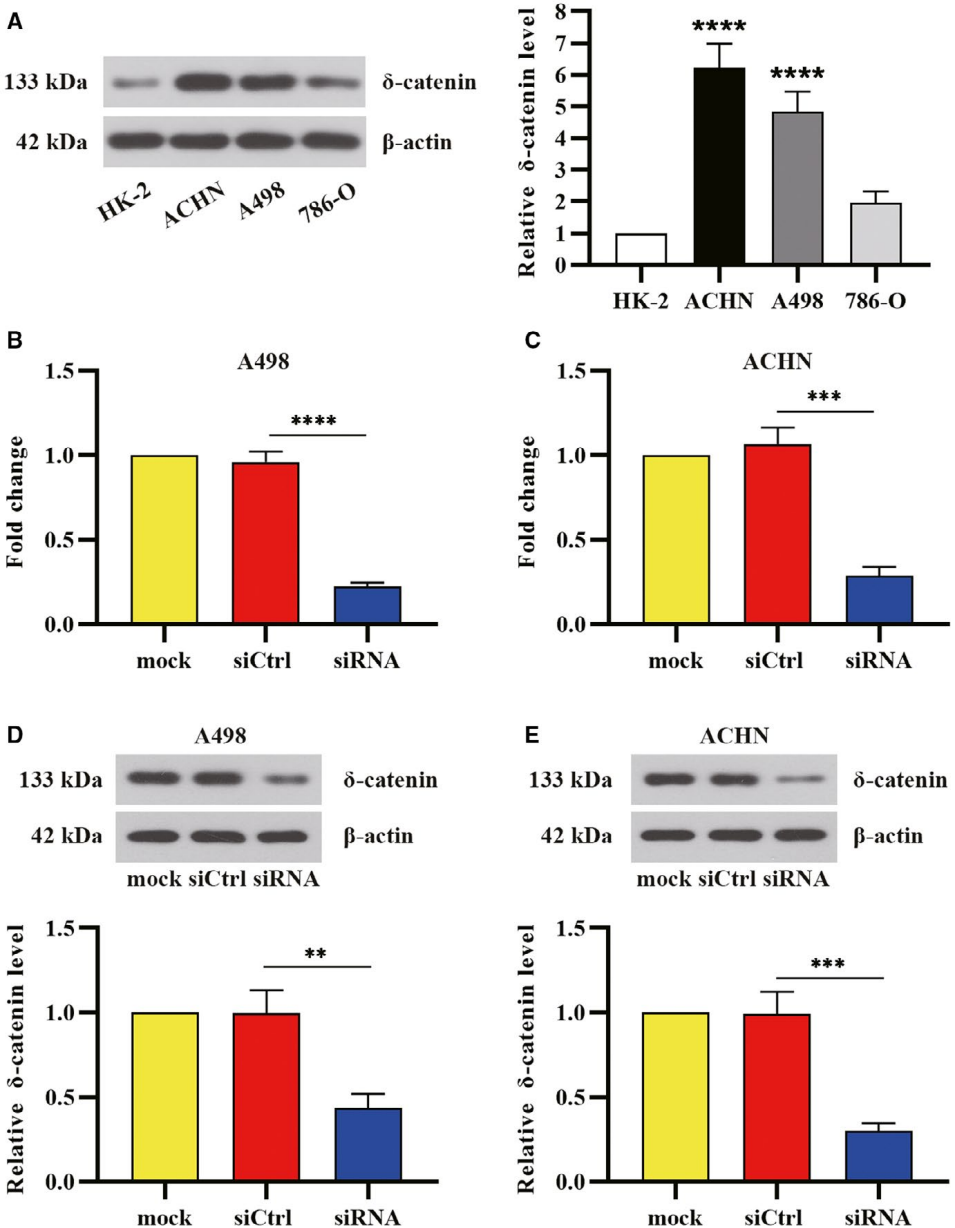
δ‐Catenin was highly expressed in human renal cancer cells. A, The expression of δ‐catenin in renal cancer cell lines tested by Western blot analysis. Renal cancer cells A498 and ACHN were treated with δ‐catenin‐specific siRNA. After 48 h transfection, B, C, δ‐catenin mRNA levels were analyzed by qRT‐PCR (n = 3). D, E, The protein levels of δ‐catenin were detected by Western blot. Data were presented as mean ± SD (n = 3) with statistic significance calculated from one‐way ANOVA for multiple comparisons (**P < .01, ***P < .001, ****P < .0001)

Next, we determined the level of δ‐catenin in normal human renal epithelial cells (HK‐2) and three different RCC cell lines (A498, ACHN and 786‐O). As expected, δ‐catenin was highly expressed in RCC cells compared with HK‐2 cells (Figure 2A). We employed specific siRNA to knock down δ‐catenin in A498 and ACHN cells showing its relatively higher expression. qRT‐PCR measurement of A498 and ACHN cells revealed the mRNA level of δ‐catenin was reduced after δ‐catenin knockdown (Figure 2B,C). The remarkable reduction of δ‐catenin protein level was also observed in RCC cells after δ‐catenin silencing (Figure 2D,E). All the data suggested that δ‐catenin expression was elevated in RCC cells.

### 3.3 δ‐Catenin promoted renal cancer cell proliferation

**Figure 3:**
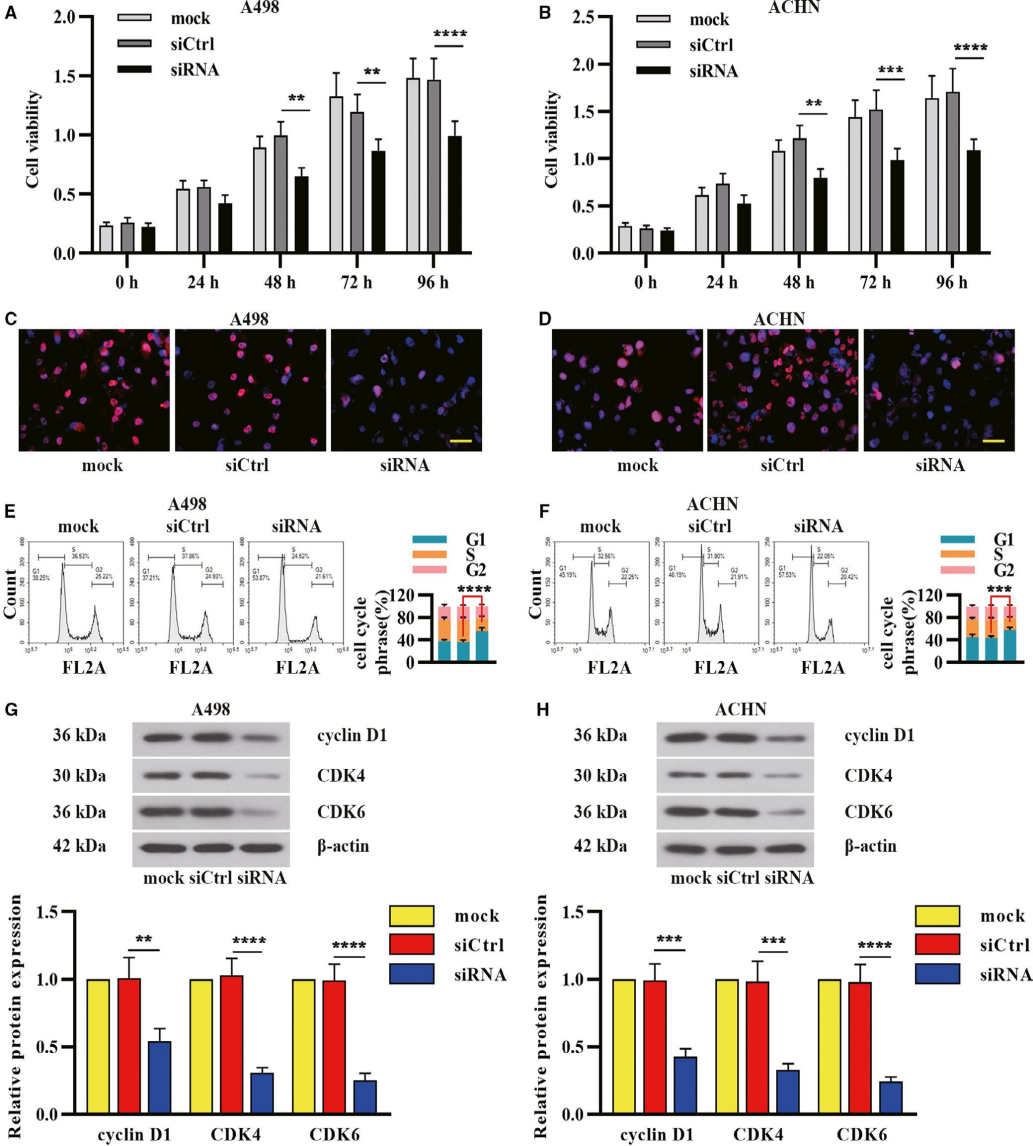
δ‐Catenin promoted renal cancer cell proliferation. δ‐Catenin‐specific siRNA was employed to silence δ‐catenin in A498 and ACHN cells. A, B, After 24, 48, 72, and 96 h of transfection, CCK‐8 assay was performed to observe cell viability. C, D, The immunofluorescence analysis of the expression of Ki67 in renal cancer cells transfected with siRNA. Scale bar, 50 μm. E, F, Cell cycle was analyzed in A498 and ACHN cells by flow cytometry. G, H, The expression level of cell cycle‐associated markers was determined by Western blot. Data in panel A, B, E, F, G, and H were shown as the mean ± SD from three separate experiments, with two‐way ANOVA for A, B, E, F, and one‐way ANOVA for G and H

Considering the correlation between high expression of δ‐catenin and poor prognosis, we investigated the biological role of δ‐catenin in RCC development. We performed CCK‐8 assay to evaluate the effect of δ‐catenin knockdown on RCC cell proliferation. As shown in Figure 3A,B, δ‐catenin knockdown resulted in decreased cell viability in A498 and ACHN cells. The immunofluorescence analysis of Ki67 also illustrated reduced proliferative capacity after δ‐catenin silencing (Figure 3C,D). We next explored whether δ‐catenin participated in RCC cell cycle regulation. We examined cell numbers in each phase of mitosis in RCC cells following δ‐catenin knockdown by flow cytometry. The results indicated that much higher percentages (53.87% in A498, 57.53% in ACHN) of RCC cells were arrested in G1 phase upon δ‐catenin knockdown (Figure 3E,F). We also determined the expression of cell cycle‐related proteins in RCC cells following δ‐catenin silencing. The expression of cyclin D1, CDK4, and CDK6 was significantly reduced after downregulating endogenous δ‐catenin (Figure 3G,H). These results strongly indicated that δ‐catenin knockdown has an inhibitory effect on proliferation of RCC cells.

### 3.4 δ‐Catenin inhibited apoptosis of renal cancer cells

**Figure 4:**
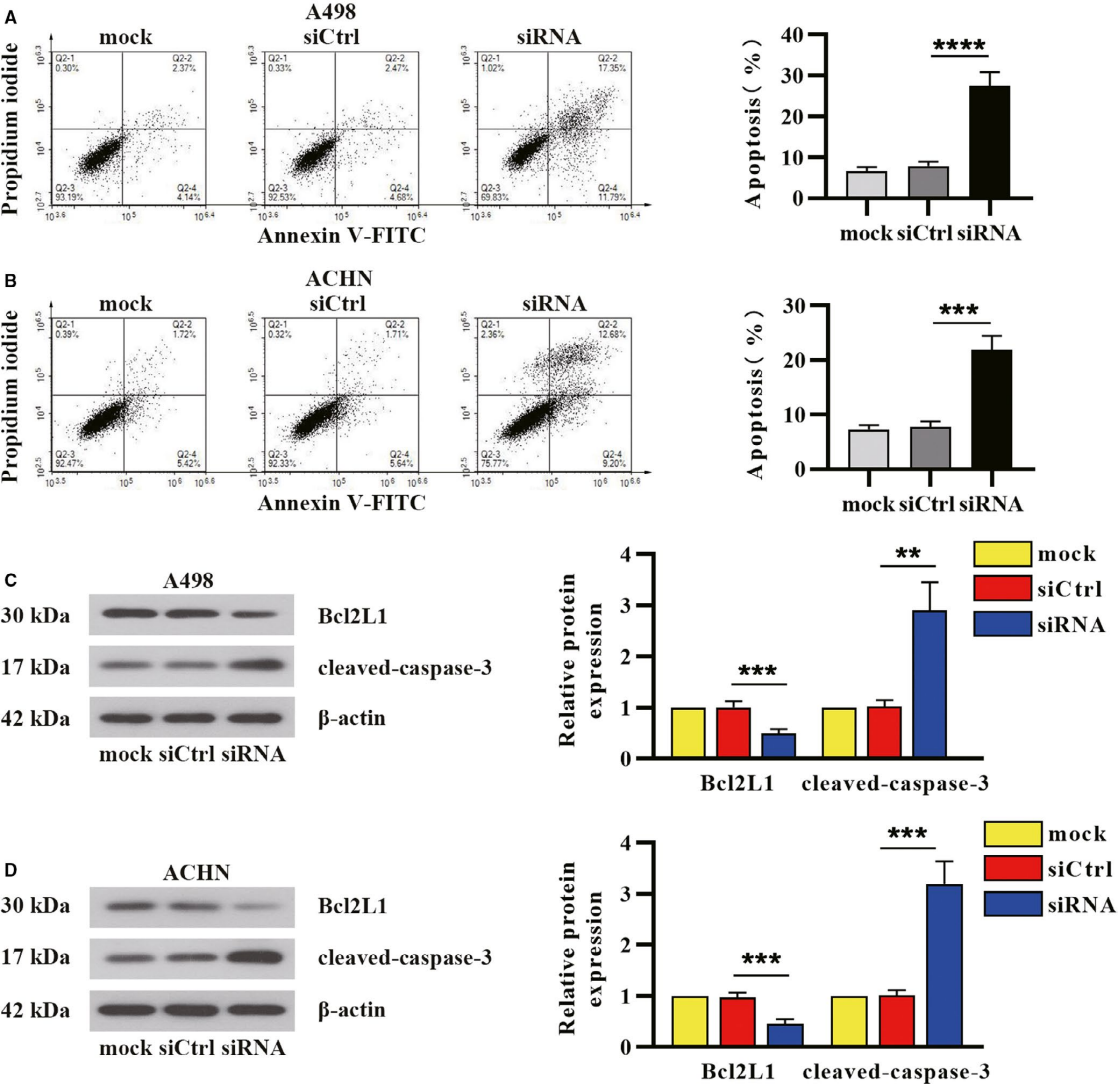
δ‐Catenin inhibited apoptosis of renal cancer cells. A498 and ACHN were transfected with δ‐catenin‐specific siRNA for 48 h. A, B, The cells were analyzed by FACS using Annexin V‐FITC/PI staining, and the percentage of apoptotic cells was calculated. C, D, The protein levels of apoptosis‐related markers in renal cancer cells following δ‐catenin knockdown were detected by Western blot. The percentage of apoptotic cells and quantitative analysis for apoptosis‐related proteins were presented as mean ± SD with one‐way ANOVA with Tukey's test for multiple comparisons. **P < .01, ***P < .001, ****P < .0001

Furthermore, we identified the effect of δ‐catenin on apoptosis of RCC cells. A498 and ACHN cells transfected with siRNA were stained with Annexin V‐FITC/PI and subjected to flow cytometric analyses. The percentage of apoptotic cells was increased in δ‐catenin‐knocked down RCC cells (Figure 4A,B). We also examined protein expression of apoptosis‐related markers by Western blot analysis. As shown in Figure 4C,D, silence of δ‐catenin led to the downregulation of Bcl2L1 (anti‐apoptotic marker) but upregulation of cleaved‐caspase 3. All these data substantiated δ‐catenin restrained the induction of apoptosis in RCC cells.

### 3.5 δ‐Catenin affected β‐catenin nuclear localization and its downstream gene expression in renal cancer cells

**Figure 5:**
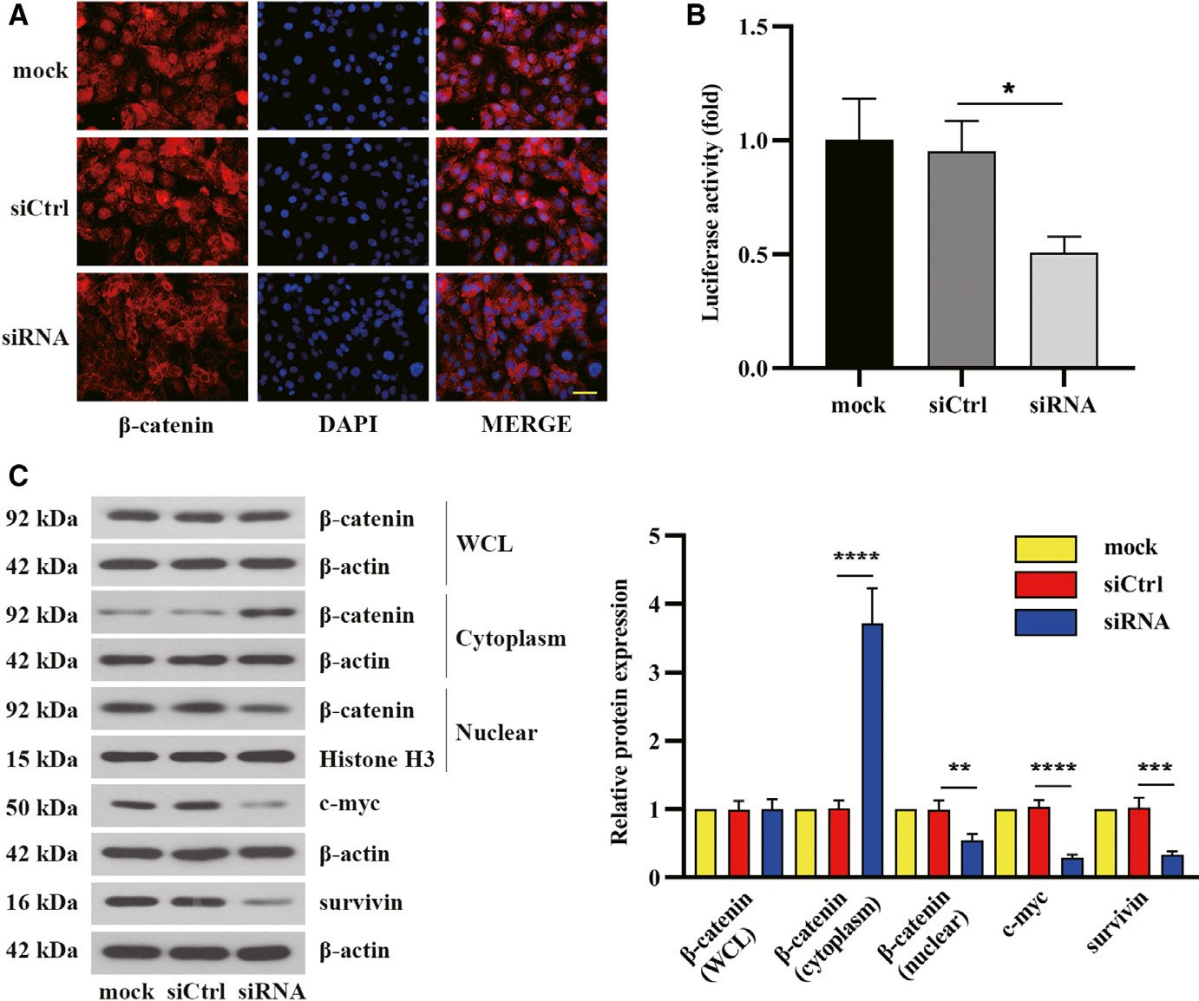
δ‐Catenin affected β‐catenin nuclear localization and its downstream gene expression in renal cancer cells. ACHN cells were treated with δ‐catenin specific siRNA for 48 h. A, The expression and localization of β‐catenin were observed by immunofluorescence analysis. Scale bar, 50 μm. B, The transcriptional activity of β‐catenin following δ‐catenin knockdown was analyzed by the TOP/FOP luciferase reporter assay. The relative luciferase activity was normalized and shown (n = 3). C, The expressions of β‐catenin in the whole cell lysate, cytoplasm, and nucleus as well as the protein levels of c‐myc and survivin were tested by Western blot. Data in panel B and C were shown as the mean ± SD based on one‐way ANOVA with Tukey's test (*P < .05, **P < .01, ***P < .001, ****P < .0001)

Considering δ‐catenin could activate β‐catenin and its downstream tumor‐associated signaling pathways to participate in the development of prostate cancer,^14^ we investigated the effect of δ‐catenin on this pathway in RCC. Immunofluorescence analysis was employed to visualize the change in nuclear localization of β‐catenin under the condition of δ‐catenin silence. As shown in Figure 5A, δ‐catenin silencing in ACHN cells led to a remarkable decrease in nuclear β‐catenin, which was further confirmed by Western blot analysis (Figure 5C). To further investigate whether the altered β‐catenin distribution interacts with TCF/LEF to activate downstream gene expression, TCF/LEF transcriptional reporter activity was measured in ACHN cells. Knockdown of δ‐catenin exhibited a lower specific TCF/LEF transcriptional reporter activity (Figure 5B), implying an alteration in the transcription co‐activator activity of β‐catenin, which caused the subsequent downregulation of downstream target gene, such as c‐myc and survivin (Figure 5C). Together, these results indicated that δ‐catenin upregulated downstream gene expression of β‐catenin by promoting β‐catenin translocation to the nucleus in RCC cells.

### 3.6 δ‐Catenin promoted tumor growth in ACHN xenograft mouse model

**Figure 6:**
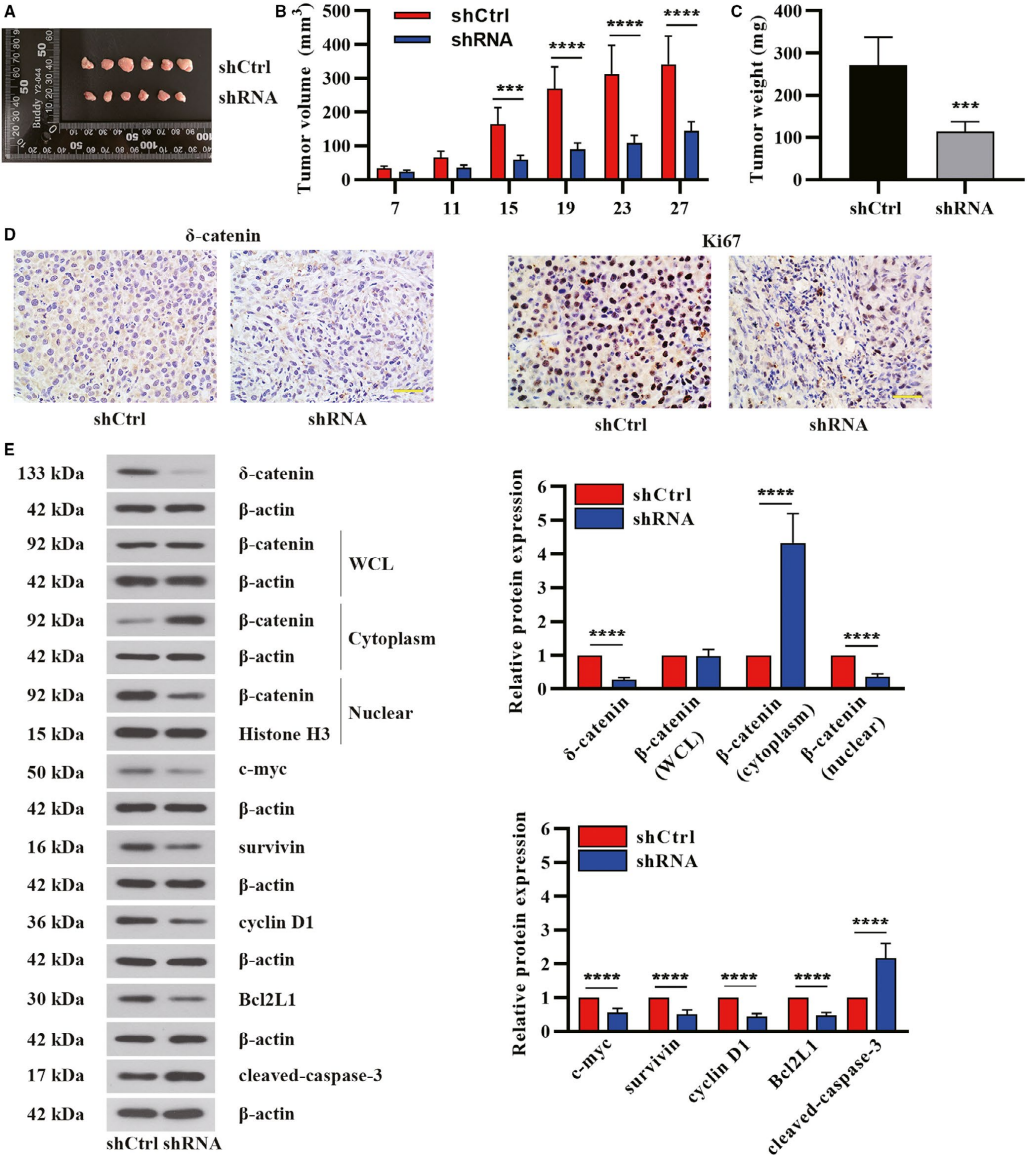
δ‐Catenin promoted tumor growth in ACHN xenograft mouse model. A, The images of tumor harvested at 27 d after transplantation of ACHN cells. B, Tumor diameters were measured at a regular interval of 4 d for up to 27 d and the tumor volume was calculated. C, Tumor weight from each group was measured after the mice were sacrificed. D, The expression of δ‐catenin and Ki67 in tumor tissue sections was tested by immunohistochemistry staining. Scale bar, 50 μm. E, The protein expression of δ‐catenin, β‐catenin (in the whole cell lysate, cytoplasm, and nucleus), c‐myc, survivin, cyclinD1, Bcl2L1, and cleaved‐caspase‐3 in tumor tissues was detected by Western blot. Data in panel B were shown as the mean ± SD based on two‐way ANOVA (n = 6). Data in C and E were presented as the mean ± SD (n = 6, two‐tailed unpaired *t* test). ***P < .001, ****P < .0001

To further detect the regulation of δ‐catenin on tumorigenesis in vivo, we examined the effect of δ‐catenin knockdown on tumor growth of ACHN cell‐derived xenograft in nude mice. The ACHN cells transfected with shCtrl plasmids and δ‐catenin shRNA plasmids were subcutaneously injected into BALB/c nude mice, which were divided into two groups (n = 6 for each group). Tumor volume was recorded over a period of 27 days and the solid tumors were photographed and weighed after the mice were sacrificed. As shown in Figures 6A‐C, knockdown of δ‐catenin markedly suppressed tumor growth in mice, compared with the shCtrl group. Moreover, immunohistochemical analysis of the tumor tissues also revealed δ‐catenin knockdown suppressed tumor growth, as evidenced by reduced Ki67 expression (Figure 6D). Furthermore, δ‐catenin knockdown restrained translocation of β‐catenin to the nucleus, as well as regulated the expression of c‐myc, surviving, cyclinD1, Bcl2L1, and cleaved‐caspase‐3 (Figure 6E). All these results validated that δ‐catenin facilitated tumor growth in vivo in RCC.

## 4 DISCUSSION

It has been proven that δ‐catenin has ectopic overexpression in lung cancer and prostate cancer and promotes tumor progression,^12^, ^13^, ^14^ but little is known about its biological role and the associated signaling pathways in RCC. Our study set out to determine whether δ‐catenin was implicated in the development of RCC. Based on the results of Western blot and immunohistochemistry, we found that the level of δ‐catenin is elevated in clinical tissue samples with RCC. Therefore, we reasoned that δ‐catenin also played a role in promoting the development of RCC. In subsequent exploration, we found that δ‐catenin affected the translocation of β‐catenin, which further modulated the expression of its downstream apoptosis‐related protein Bcl2L1, cell cycle‐associated marker cyclin D1 as well as tumorigenic gene c‐myc and survivin, thereby facilitated proliferation and suppressed apoptosis in RCC. Moreover, the RCC cell xenograft mouse model revealed knockdown of δ‐catenin restrained tumor growth in vivo, which was concordant with our in vitro findings.

Researches conducted by Zhang et al^12^ have assessed the clinical significance of δ‐catenin and its correlation with clinicopathological factors in a cohort of 115 specimens with non‐small‐cell lung cancer (including 65 cases with follow‐up records and 50 cases with paired lymph node metastases lesions). The results demonstrated that high expression of δ‐catenin could be observed in adenocarcinoma. Positive expression of δ‐catenin was enhanced in lymph node metastasis lesions, compared with corresponding primary tumors. The elevated δ‐catenin expression was significantly correlated with higher pTNM stage and lymph node metastasis. Furthermore, patients harbouring tumors with δ‐catenin positive expression had significantly shorter postoperative survival period than patients harboring tumors with negative expression. Besides, it has been reported that increased δ‐catenin expression in estimated 85% of prostate cancer based on tissue samples (composed of 90 cases with prostate cancer and 90 cases with benign prostate). Meanwhile, the enhanced δ‐catenin expression was concerned with increased Gleason scores.^29^ Consistent with these reports, a significant finding of our study is that RCC patients showed high expression of δ‐catenin and it is correlated with some clinicopathological factors, such as pTNM stage, tumor stage and lymph nodes metastasis. It gave evidence that positive expression of δ‐catenin was relevant to poor prognosis in RCC. δ‐Catenin might be established as a potential prognostic factor and a therapeutic target of RCC.

In this study, we found δ‐catenin promoted RCC cell proliferation by altering G1 to S phase transition in cell cycle. Furthermore, δ‐catenin restrained apoptosis of RCC cells through upregulating Bcl2L1 (a long isoform of Bcl2) that protects cells from undergoing apoptosis.^30^ In the past decades, a large and growing body of literature has shown that cyclin D1 was induced by hyperactivated β‐catenin, and thereby led to G1 phase progression and proliferation of tumor cell.^31^, ^32^, ^33^, ^34^ On the other hand, Rosenbluh et al stated that β‐catenin forms a complex with YAP1 and transcription factor TBX5 which binds to the promoter of BCL2L1, thereby directly regulating BCL2L1 expression at transcriptional level.^35^ Taken together, aberrant cyclin D1 and BCL2L1 upregulation take place when the Wnt/β‐catenin pathway is activated. Combined with our investigation about the effects of δ‐catenin on proliferation and apoptosis in RCC cells, we deduced that δ‐catenin may exert these functions by regulating β‐catenin in RCC development. As expected, our data showed that δ‐catenin knockdown suppressed β‐catenin nuclear localization and subsequent alteration of Bcl2L1 and cyclin D1 level in both ACHN cells and ACHN‐derived xenograft tumor tissue.

Given that δ‐catenin affected subcellular β‐catenin distribution and activated the downstream effectors of β‐catenin in prostate cancer,^14^ we explored whether δ‐catenin played the same role in RCC progression. Figure 5 elucidated δ‐catenin realized the tumor‐promoting function in renal tumorigenesis by altering β‐catenin translocation. Notably, silencing of δ‐catenin prevented β‐catenin from translocating to the nucleus, which resulted in the repression of β‐catenin‐TCF/LEF‐mediated downstream effector activation. Knockdown of δ‐catenin in RCC cells revealed a markedly decreased expression in β‐catenin target genes such as Bcl2L1, cyclin D1, c‐myc, and survivin.

In our study, we used RNA interference (RNAi) including transient siRNA and stable shRNA for loss‐of‐function studies to investigate the role of δ‐catenin in RCC development. One major drawback of these means is the limited utility, as evidenced by δ‐catenin‐silenced cells still could grow and preserve tumorigenesis capacity. In future research, the inducible system (such as the tetracycline‐controlled operator system) should be combined with RNAi, which could reveal the direct effects caused by the loss of δ‐catenin via comparing noninduced and induced cells, thereby accurately evaluating the crucial role of δ‐catenin in RCC cell growth and apoptosis.

In summary, our study is the first to report δ‐catenin was overexpressed in tissues of RCC patients and its correlation with pTNM stage, tumor stage, and lymph nodes metastasis. In addition, knockdown of δ‐catenin can obviously decrease the proliferation capacity and induce apoptosis of RCC cells by affecting β‐catenin nuclear localization and its downstream target gene expression. Therefore, our results illustrated that δ‐catenin might be a prognostic marker and a promising therapeutic target in RCC.

## CONFLICT OF INTEREST

The authors declare that they have no competing interests.

## AUTHOR CONTRIBUTIONS

Yongsheng Song conceived and designed the study. Ju Lincheng performed the experiments and analyzed the data. Shan Liping wrote the paper. Yinbo obtained funding. All authors have read and approved the final manuscript.

## Data Availability

All data generated or analyzed during this study are included in this published article.
