# Supplementary figures and images for: δ‐Catenin regulates proliferation and apoptosis in renal cell carcinoma via promoting β‐catenin nuclear localization and activating its downstream target genes
Source: Cancer Med. 2020 Jan 28;9(6):2201–12. doi: 10.1002/cam4.2857 (PMC7064116; doi:10.1002/cam4.2857)

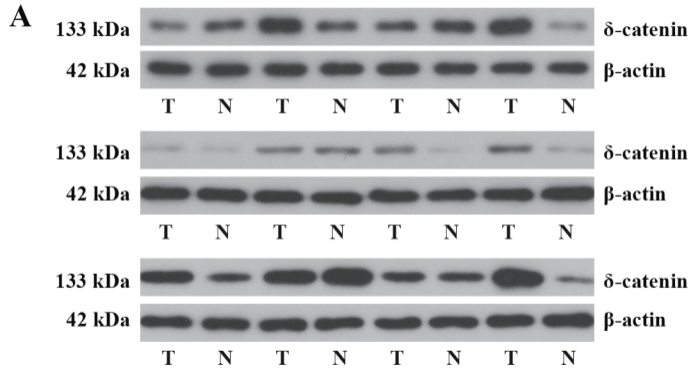

Supplement: Supplementary file 1 [file CAM4-9-2201-s001.pdf]
